# Spatial Analyses of Oral Polio Vaccine Transmission in an Community Vaccinated With Inactivated Polio Vaccine

**DOI:** 10.1093/cid/ciy622

**Published:** 2018-10-30

**Authors:** Christopher I Jarvis, Jonathan Altamirano, Clea Sarnquist, W John Edmunds, Yvonne Maldonado

**Affiliations:** 1 London School of Hygiene and Tropical Medicine, United Kingdom; 2 Medical Research Council London Hub for Trials Methodology Research, United Kingdom; 3 Stanford University School of Medicine, California

**Keywords:** cluster randomized trials, oral polio vaccine, polio, spatial

## Abstract

**Background:**

Understanding the spatial dynamics of oral polio vaccine (OPV) transmission will improve resource targeting. Mexico provides a natural laboratory, as it uses inactivated polio vaccine routinely as well as OPV bi-annually.

**Methods:**

Using geospatial maps, we measured the distance and density of OPV vaccinees’ shedding in the areas nearest to unvaccinated households in 3 Mexican villages. Comparison of transmission to unvaccinated households utilized a mixed effects logistic regression with random effects for household and time, adjusted for age, gender, area, and running water.

**Results:**

The median distance from an unvaccinated household to its nearest OPV-shedding household was 85 meters (interquartile range, 46–145) and the median number of vaccinees shedding OPV within 200 m was 3 (2–6). Transmission to unvaccinated households occurred by day 1. There was no association (odds ratio [OR] 1.04; 95% credible interval [CrI] 0.92–1.16) between the distance from OPV shedding and the odds of transmission. The number of OPV vaccinees shedding within 200 m came close to a significant association with unvaccinated transmission (OR 0.93; CrI 0.84–1.01), but this was not the case for households 100 or 500 m apart. Results were consistent across the 3 villages.

**Conclusions:**

Geospatial analysis did not predict community transmission from vaccinated to unvaccinated households, because OPV use resulted in rapid, low transmission levels. This finding supports the global cessation of OPV.

The goal of global polio eradication may soon be reached. In 2016, there were 37 cases of wild-type polio, a decrease from the 87 cases in 2015 [1, 2]. Currently, only Pakistan and Afghanistan continue to have endemic transmission of wild-type serotype 1 polio, and Nigeria reported 4 cases in September 2016 [2]. The success of the polio eradication effort can be largely attributed to the use of oral polio vaccine (OPV) in developing countries, due to its low cost, easy administration, and ability to confer passive immunization to contacts, presumably via fecal-oral infections. However, the risks from OPV use are now complicating eradication efforts. Vaccine-associated paralytic polio is a rare, adverse reaction to OPV administration, occurring in every 900000 doses [3]. Of more concern, prolonged circulation of OPV, primarily in communities with suboptimal sanitation, can result in OPV mutation and neuroreversion, leading to circulating vaccine-derived poliovirus (cVDPV). In addition, cVDPVs studied in Nigeria were found to be as virulent as wild-type polio [3].

To date, the spatial characteristics of OPV transmission are not well characterized. Understanding the pattern and extent of geographic variation in OPV circulation could help to predict and prepare for the risk of OPV reintroduction, especially in undervaccinated communities. Areas of increased risk could be detected, and resources accordingly deployed to reduce or prevent prolonged OPV circulation. In the current global setting of polio transmission, this is particularly important for serotype 2, which was declared eradicated as of September 2015 by the World Health Organization [4].

In this study, we had the opportunity to identify household and community transmission of OPV in Mexico, where inactivated polio vaccine (IPV) is provided for routine immunization at 2, 4, 6, and 18 months of age and OPV is only administered in 2 National Health Weeks (NHW) to children 5 years of age and under. We were able to investigate spatial transmission at a household level within 3 villages that received different levels of OPV vaccinations during the February 2015 NHW. Furthermore, we collected stool samples that allowed for the detection of OPV transmission from vaccinated children to their households and community contacts. Spatial analyses of polio transmission have been performed as far back as 1967 [5]; however, most involved aggregated case data over large areas of several kilometers [6–8]. This study allowed an investigation at a local level of what happens in a community when the OPV vaccine is introduced.

In this paper, we explore 2 aspects of between-household transmission of polio. First, we consider whether living near someone who is shedding poliovirus affects an individual’s chance of shedding poliovirus. Second, we determine how the number of people shedding near an unvaccinated contact impacts that individual’s chance of acquiring and shedding poliovirus. We refer to the distance and density of shedding, collectively, as proximity to shedding.

## METHODS

### Study Design

The study has already been described in detail elsewhere [9] but, in brief, this was a prospective, cluster-randomized trial in 3 indigenous localities in Orizaba, Veracruz, Mexico (Capoluca, Campo Grande, and Tuxpanguillo). Within each community, approximately 150 households were enrolled in this study, and each community cluster received a different amount of OPV coverage as part of the study: 70% of enrolled households in Capoluca, 30% in Campo Grande, and 10% in Tuxpanguillo. When enrollment began in February 2015, 155 households were randomized to receive OPV out of 466 households included across the 3 localities. No other households in any of the 3 communities received OPV until the May 2015 NHW. Only 1 child from each of the 155 households received OPV. Inclusion criteria for household enrollment was the presence of a child <5 years old who had an up-to-date IPV vaccination record and was eligible to receive OPV. All adult participants consented to participation, and the guardians of minors consented for minors to participate. Exclusion criteria for children <5 included presentation with illness (febrile, diarrhea, or respiratory); an immunodeficiency caused by AIDS, disease, or medication; a recent blood transfusion; and prior adverse reactions to OPV. Exclusion criteria for all other participants was refusal to participate or a change in residence during the study period. Within our study population, global positioning system (GPS) coordinates were collected from 423 households: 137 of the vaccinated households and 286 unvaccinated households. As a result, only the shedding and transmission results from these participants were considered in this analysis.

After enrollment, 10 stool samples were scheduled for collection from each member of all enrolled households: 1 baseline sample before vaccination and 1 sample each at 1, 4, 7, 10, 14, 21, 28, 51, and 71 days after vaccination. During each visit, health information, travel and visit details, and records for any vaccines received during the study period for children <5 were collected via follow-up surveys. Exclusion criteria for follow up were individuals that refused to participate, changes in residence during the study period, or absence during follow-up visits.

Viral ribonucleic acid (RNA) was extracted from frozen stool samples utilizing the MagNA Lyser (Roche) and KingFisher Duo Prime (Fisher Scientific), using the bacteriophage MS2 as an internal control for extraction efficiency. Viral RNA then underwent quantitative, reverse-transcription polymerase chain reaction (rt-QPCR) in order to detect and quantify any Sabin OPV present in the samples. The probes and primers were adopted and adapted from Kilpatrick et al. [10] and the Centers for Disease Control protocol for polio quantitative-PCR. Samples were run in triplicate and a sample was considered positive if two-thirds of reactions had a cycle threshold (Ct) <37. Positive samples were re-run to minimize false positives and, if positive, the RNA was Sanger-sequenced again for confirmation.

### Sample

We used 2 types of participants from the study: vaccinated individuals and individuals who lived in unvaccinated households, referred to here as unvaccinated individuals. We were interested in the outcomes of unvaccinated individuals and their proximity to vaccinated individuals. Therefore, vaccinated participants were not analyzed directly; instead, we created spatial variables for unvaccinated participants by comparing the locations of vaccinated and unvaccinated households. For instance, we measured the distance from an unvaccinated person to their nearest vaccinated household. We did not include unvaccinated people who lived in vaccinated households, as the focus of our analyses was between-household transmission. Within-household transmission is being analyzed separately [11]. Vaccinated individuals were treated as sources, because we could be more certain their shedding was due to the OPV and not transmission from other participants.

### Descriptive Analyses

Maps were used to visualize the spatial distribution of vaccination and shedding. We represented the density and location of vaccinated shedding over time using contour plots and overlaid the position of unvaccinated participants.

The distributions of key variables were assessed graphically and through the calculation of summary statistics. Spatial variables were summarized using medians and interquartile ranges, as they were skewed. Coverage of the vaccine was considered important to adjust for, as higher coverage led to both a smaller distance to the nearest shedding household and a higher proportion of shedding.

### Statistical Analyses

#### Outcomes

Stool samples were collected from participants at 10 points in time. We only analyzed data from the first 28 days of the study, as almost all the shedding occurred before this point. We first looked at the presence of poliovirus in stool samples and the proximity of unvaccinated partipants to vaccinated participants’ shedding at any time point in the first 28 days. Second, we considered the presence of poliovirus and the proximity of households at each given point in time.

#### Spatial Variables

For the first analysis, the outcome was a binary variable for shedding at each point in time throughout the study period. We used a spatial variable approach, as identified in a recent systematic review [12]. The approach includes a covariate for spatial proximity in a regression model and is similar to previous spatial analyses of mosquito bed-net trials [13, 14]. We measured each unvaccinated participant’s proximity to any vaccinated participant that was shedding, aggregated over the study period. Proximity was measured in 2 ways: the distance to the nearest vaccinated, shedding household and the number of vaccinated individuals shedding within 100, 200, 500, and 1000 m. Sensitivity analyses were performed, restricting the data to the first 14 days to reduce the risk of shedding being recorded due to secondary transmission.

For the second analyses, we calculated the same spatial variables, but within each point in time; therefore, the distance to the nearest vaccinated, shedding household could change depending on the study day. We also calculated the spatial variables using calendar days after initiation of the NHW OPV administration, and found no differences in the outcomes. In addition to this, we examined whether the amount of the viral load surrounding an unvaccinated household affected the likelihood of shedding. Here, we used the sum of the log viral load, where a greater number represents a higher viral load and a lower number a lower viral load.

#### Modelling

The study is a cluster-randomized trial with a hierarchical structure, giving multiple observations per individual per household. Mixed-effects logistic regression with a random effect for household was used to assess the association of the distance and density of vaccinated shedding with unvaccinated shedding over the 28-day period. A mixed-effects logistic regression with a random effect for household and an autoregressive lag 1 random effect for study day was used to look at the impact of the distance and density of OPV shedding and viral loads. The autoregressive random effect allows the previous study period to provide information to the next period. We adjusted for participant age and gender and for access to running water within the household.

The spatial variables were included as continuous variables. Non-linearity was explored using quadratic terms and treating the variables as categorical; there was no suggestion of non-linear effects. The analyses were repeated by Sabin type: this increased the sparsity of the data and those results are not included, as they were consistent with the main analyses. The models were fitted using integrated, nested Laplace approximation, [15] and all analyses were performed using R [16]

## RESULTS

### Descriptive

There are 1145 unvaccinated individuals included in the analyses, from 286 households. The age distribution was positively skewed, with a median age of 17 years and a range from 1 month to 95 years. Female participants made up 58.1% of the study. Further details, stratified by community, can be seen in Table 1. Unvaccinated individuals provided 10059 stool samples in total: 978 (85.4%) people contributed 8 or more out of a possible 10 samples and 57 (5.0%) individuals contributed only 1 sample. There was no missing data for the variables of age, running water in household, and gender, and the only missing data was the omission of stool samples. If we assume each participant could have provided 10 stool samples, then we observed 87.8% of the 11450 potential samples for unvaccinated individuals.

**Table 1.** Characteristics of Unvaccinated Individuals by Coverage Area

	Coverage Area			Total
	70% Vaccinated	30% Vaccinated	10% Vaccinated	
Households with GPS, n	126	136	161	423
Vaccinated households, n	80	40	17	137
Unvaccinated households, n (%) (%)	46 (36.5%)	96 (70.6%)	144 (89.4%)	286 (67.6%)
**Unvaccinated participants, n**	161	396	588	1145
Shedding, n (%)	24 (14.9%)	32 (8.1%)	24 (4.1%)	80 (7.0%)
Age in years, median (IQR)	12.2 (4.0 to 27.0)	17.0 (4.0 to 30.2)	18.0 (4.2 to 33.0)	17.0 (4.1, 31.0)
Female, n (%)	98 (60.9%)	223 (56.3%)	344 (58.5%)	665 (58.1%)
Running water, n (%)	142 (88.2%)	343 (86.6%)	521 (88.6%)	1006 (87.9%)
Samples provided, n	1294	3202	5563	10059
Positive samples, n (%)	30 (2.3%)	33 (1.0%)	26 (0.5%)	89 (0.9%)

Abbreviations: GPS, global positioning system; IQR, interquartile range.

In unvaccinated individuals, there were 89 (0.9%) positive samples, which came from 80 (7.0%) individuals; only 6 people contributed more than 1 positive sample. The number of positive samples varied over time, with 24 positive samples observed at day 7, 2 at day 10, and 8 at day 14. More details at each point in time by locality can be found in the Supplementary Material.

**Figure 1.**
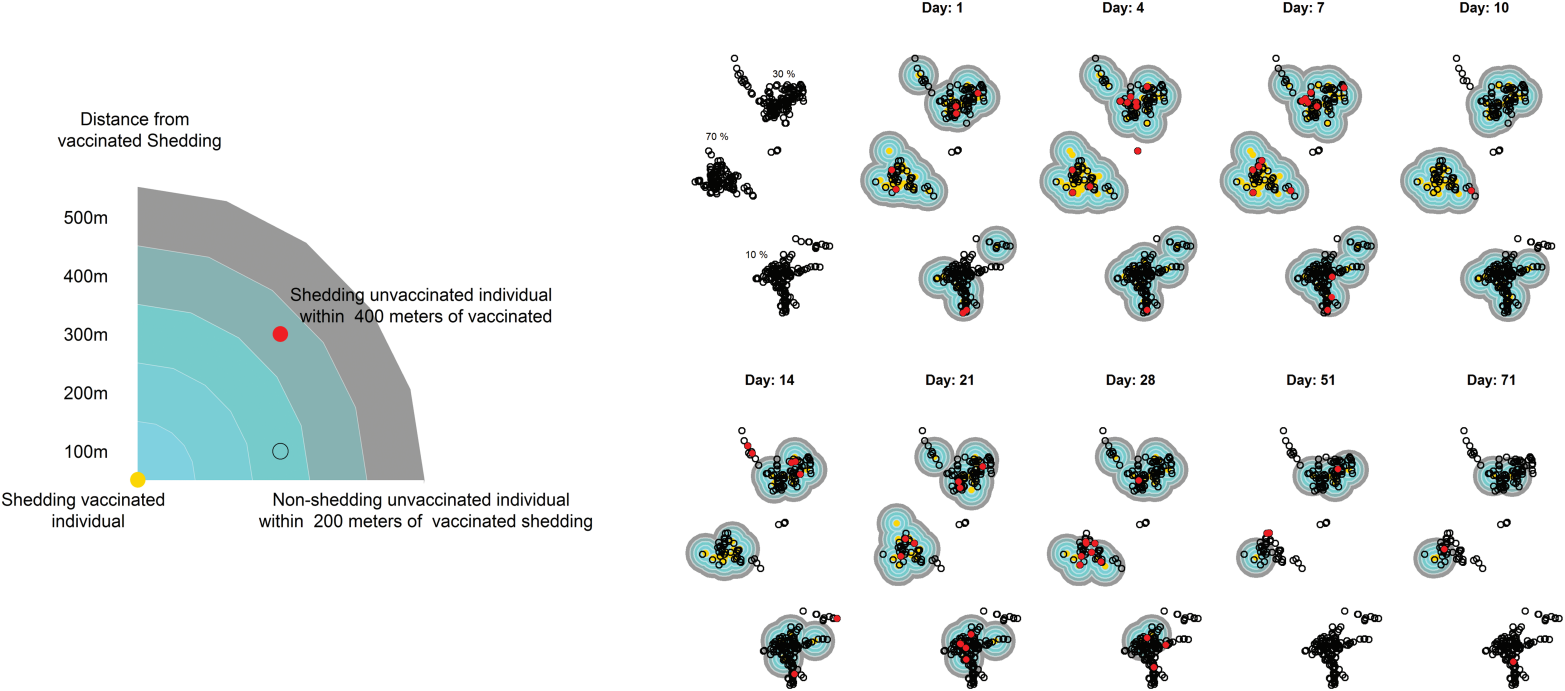
Spatial mapping of oral polio vaccine transmission shedding over time.

In the original study, 155 children were vaccinated. Of these, 137 (88.4%) had GPS data that was used to calculate the spatial variables; the remaining 18 did not have GPS data and were not used in the analyses, as we do not know their locations. The locations of the households, as well as the participants intervention and outcome statuses, are displayed in Figure 1. The median distance to vaccinated households for unvaccinated individuals was 77.6 m (interquartile range 42.0 to 126.1 m). All unvaccinated households were within 826.4 m of a vaccinated household at some point in the study, and no household was closer to shedding in any village other than in their own.

The vaccinated children provided 1237 samples, of which 342 (27.6%) were positive. There were 108 vaccinated children who shed OPV at any point in the study.

**Figure 2.**
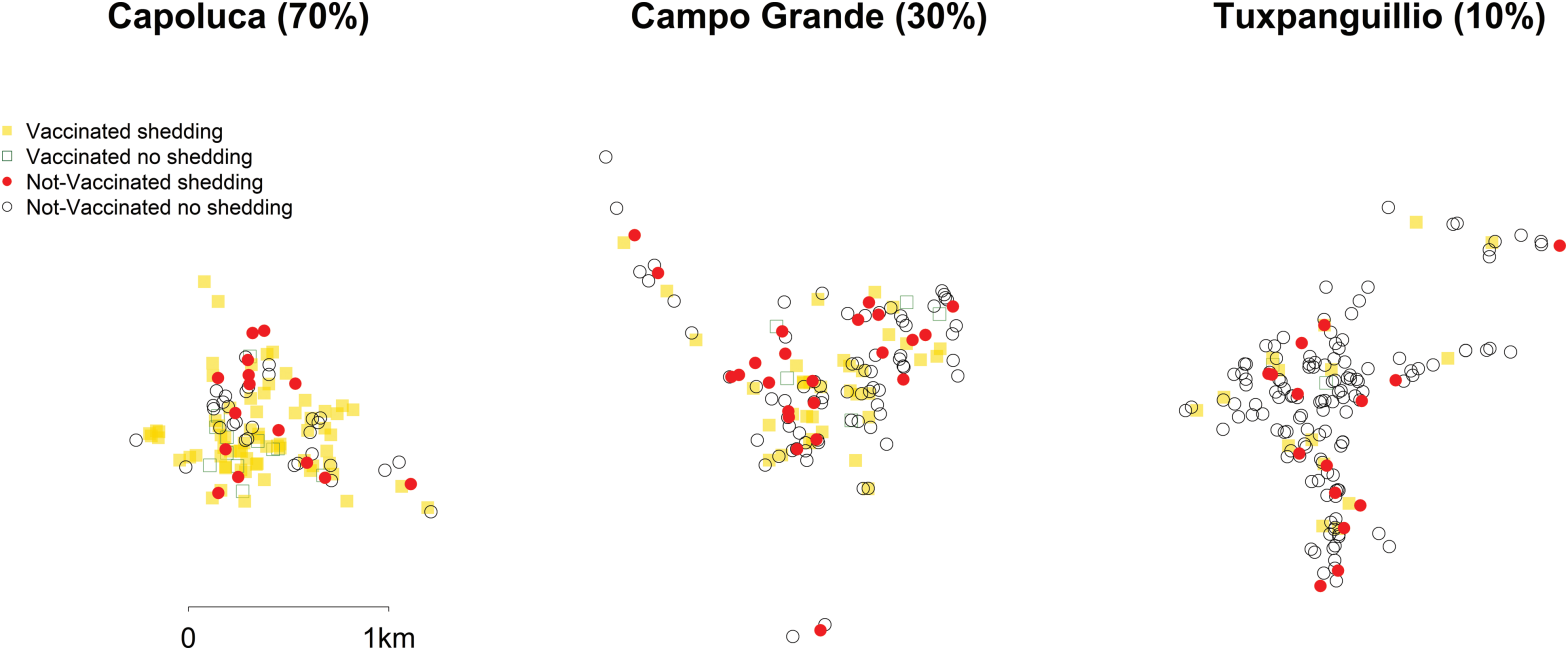
Maps of study areas.

An analysis of the samples collected at baseline confirmed that no children <5 were shedding before the study began. Shedding began quickly, and between-household transmission occurred on the first study day in some cases. In addition, the vaccinated children tended to shed more consistently throughout the study, with 36 (26.3%) individuals providing 4 or more positive samples and 2 individuals providing 8 positive stool samples. This can be seen in Figure 2, where the contours are present early on and are consistent throughout the study. Unvaccinated shedding occurred early in the study, but the location of shedding was more variable over time.

### Spatial Variables

**Figure 3.**
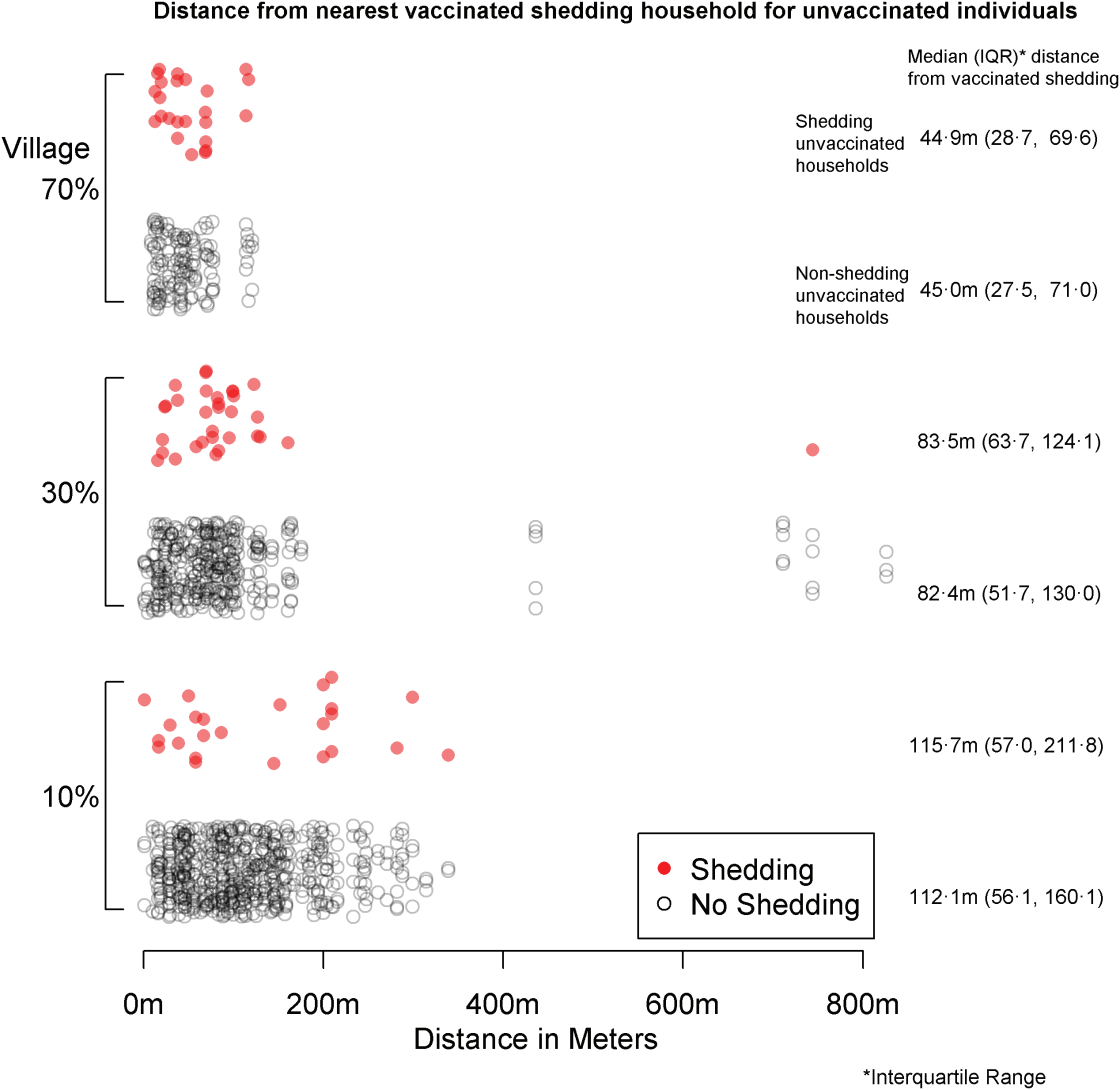
Distance from nearest vaccinated shedding household for unvaccinated individuals.

**Figure 4.**
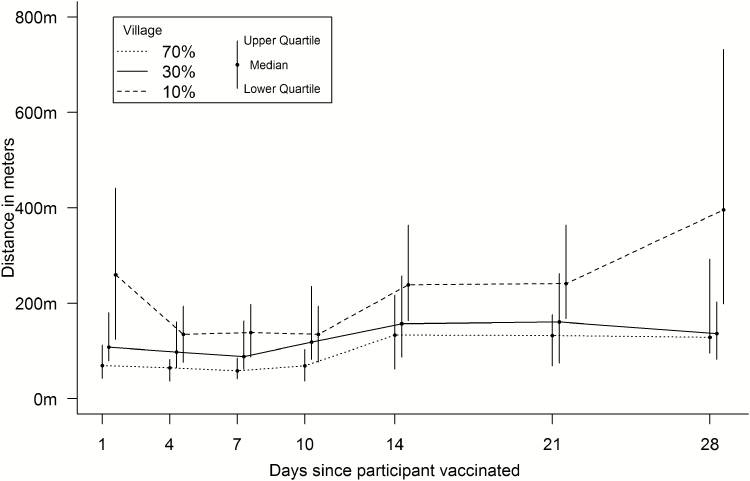
Median distance from vaccinated shedding over time.

The median distance from unvaccinated participants to vaccinated, shedding participants during the first 28 days was 85.0 m (46.0 to 145.0 m), with a breakdown by village given in Figure 3. The median distance at any given time varied over the study period, ranging from 126.1 m on study day 4 to 1626.3 m on study day 71, when there were very few cases of vaccinated shedding (Figure 4). The median number of shedding individuals within 200 meters was 3 (2 to 6); further distances are shown in Table 1. There were no discernible patterns for proximity after stratifying by outcome and locality; these summary measures are presented in Table 2.

**Table 2.** Spatial Characteristics Comparing Unvaccinated Shedding with Unvaccinated Non-Shedding Individuals

Unvaccinated	N	Distance to Nearest Vaccinated		Number of Vaccinated Shedding Households Within:			
		Household	Shedding Household	100 m	200 m	500 m	1000 m
70% Coverage		Median (IQR)					
Positive	24	42.5 (19.7, 69.6)	44.9 (28.7, 69.6)	2.5 (2, 5)	10 (7, 19)	54 (43, 61)	78 (77, 79)
No Shedding	137	41.8 (18.5, 57.7)	45 (27.5, 71)	3 (2, 5)	10 (8, 16)	58 (43, 62)	78 (77, 78)
30% Coverage							
Positive	32	78.8 (53.6, 99.5)	83.5 (63.7, 124.1)	1 (1, 2)	6 (1.8, 8)	22.5 (16, 25.2)	38 (37, 39)
No Shedding	364	71.3 (37.9, 99.8)	82.4 (51.7, 130)	1 (1, 2)	5 (2, 8)	22 (13.8, 26)	38 (36, 39)
10% Coverage							
Positive	24	115.7 (56.1, 209.4)	115.7 (57, 211.8)	0.5 (0, 1)	2 (0, 3)	6 (3.8, 10)	15 (12.2, 15)
No Shedding	564	99.7 (55.3, 150.2)	112.1 (56.1, 160.1)	1 (0, 1)	2 (1, 3)	8 (4, 10)	15 (14, 16)

All distances shown are in meters.

Abbreviation: IQR, interquartile range.

### Models

After adjusting for age, gender, and access to running water, there was very little suggestion (odds ratio [OR] 1.15; 95% credible interval [CrI] 0.86–1.46) of an association between distance (per 100 meters) from a vaccinated, shedding household and the odds of shedding for an unvaccinated individual. This was consistent when considering non-linear effects of distance. Incorporating time into the analysis with an autoregressive lag of 1 resulted in comparable results, with an OR of 1.04 (CrI 0.92–1.16). There was also no indication that the density of vaccinated household shedding within 200 m of an individual affected their odds of shedding (OR 0.99; CrI 0.95–1.04). When including time, there was some suggestion that the number of shedding, vaccinated individuals within 200 m may have had some effect on the shedding of unvaccinated individuals (OR 0.93; CrI 0.84–1.01). Results for other distances are displayed in Table 3. Restricting the analysis time to 14 days instead of 28 gave consistent results. The viral loads of individuals surrounding a person did not appear to have an association with the odds of shedding (results are presented in the Supplementary Material).

**Table 3.** Model Considering Effects of Spatial Variables on Unvaccinated Shedding

	Aggregated Over Time	Including Time
Distance to nearest vaccinated:	OR (95% CrI)	
Household, per 100 m	1.15 (0.86–1.45)	...
Shedding household, per 100 m	1.15 (0.86–1.46)	1.04 (0.92–1.16)
Number of vaccinated shedding:		
100 m	1.00 (0.87–1.13)	1.02 (0.82–1.21)
200 m	0.99 (0.95–1.04)	0.93 (0.84–1.01)
500 m	0.99 (0.98–1.01)	0.98 (0.96–1.01)
1000 m	1.0 (0.97–1.03)	1.0 (0.98–1.02)

Model adjusted for age, gender and running water in household.

Abbreviations: CrI, credible interval; OR, odds ratio.

## DISCUSSION

Through visualization of transmission onto maps, we were able to determine the dynamics of geospatial OPV transmission in a community with primary, IPV-induced immunity. We found that shedding due to the introduction of an oral polio vaccine (OPV) occurred rapidly and was associated with between-household transmission on the first day of OPV vaccination. We found little evidence to suggest that living nearer to a household with a person who was shedding OPV affected the likelihood of shedding OPV, up to the village dimensions of 850 meters. Indeed, there were no statistical differences in OPV acquisition among unvaccinated individuals based on their distance from vaccinated individuals. In addition, the threshold for OPV dispersion appeared to be low: between-household transmission in the 10% and 70% vaccination coverage communities were similar and, therefore, only a small amount of OPV appeared to be needed for community transmission of OPV. This raises important implications regarding the impact of using OPV in future outbreaks and vaccination campaigns, especially as the transmission of OPV would usually be undetected, at least in highly-vaccinated communities.

There are several strengths of this study. First, the study included a large amount of individual-level data, with multiple observations per person. To the authors’ knowledge, previous spatial analyses have only been conducted at an aggregated level on wild-type polio, where the detection of cases was not through stool samples. As no other children were vaccinated with OPV in these communities until May 2015, we know the precise sources of OPV in these communities during the study period, giving us insight into what happens when OPV is administered a single time. Second, we were also able to consider the impact of variation of coverage, as 3 separate villages had 10%, 30%, and 70% vaccinated cover of children. Third, as a requirement of the study was that all vaccinated children had up-to-date IPV vaccinations, this data mimics the transmission environment in future settings, as the Polio Endgame requires at least 1 dose of IPV in routine immunization schedules globally [2].

Using household location to represent a person’s location is, at best, an imprecise average of their movement throughout the day. Furthermore, it necessitates grouping people who live together to the same location. Information from contact tracing might have been useful to measure the proximity of individuals, but might not be available in practice. It seems clear that the transmission of polio is not purely spatial; when only household location is available, it appears to have a limited ability to predict transmission.

We tried to minimize any misclassification of within-household transmission as between-household transmission by only using vaccinated individuals as point sources and unvaccinated households in the analyses. We excluded individuals who were unvaccinated and living with vaccinated individuals. Misclassification cannot be removed entirely, due to the propagation of between- and within-household transmission. We also attempted to reduce the number of false positives for stool samples by using a 2-step laboratory process to identify OPV by RT-QPCR; however, it is likely that there were small numbers of false negative and false positive samples. However, given the large number of individuals and stool samples collected, it is unlikely that a small number of incorrectly-identified samples would have affected the conclusions of this paper.

Our results show that vaccinated children shed as early as 1 day post-vaccination. This result is supported by prior OPV trials, where most vaccinated children shed within 1 week of vaccination [17–19]. That OPV can be transmitted to the contacts of vaccinated children has also been well-documented in these trials. Low levels of transmission also occur as quickly as 1 week after vaccination, as shown by transmissibility trials from the 1960s, the results which are corroborated by more recent work in Zimbabwe looking at human immunodeficiency virus–infected mothers with OPV-vaccinated children [17–20]. However, these studies collected samples on a weekly basis. Our samples were collected with more granularity, and show that between-household transmission occurred within 1 day of vaccination, even in the community with 10% OPV coverage.

Household locations and spatial distribution appear to have limited use in predicting the transmission of poliovirus shedding. Therefore, in order to understand how OPV shedding occurs within a community, alternative information, such as contact patterns, should be analyzed. The mechanism for predicting shedding is not well understood, including the role of number of prior IPV and OPV doses and the viral loads seen after vaccination.

The use of a live poliovirus vaccine results in rapid dispersion and persistent transmission of the poliovirus throughout a community, up to at least 71 days. The only way to avoid this is to not use OPV or to have strong controls around those vaccinated, such as quarantine or strict hygiene protocols. At present, what we observed in this study would not be detected through any clinical screening, since all transmissions were asymptomatic and detected by the analyses of prospectively-collected stool samples. Therefore, better methods, such as the collection and analysis of sewage samples, are critically needed to ensure shedding has stopped within a community. Without this, any conclusions about the eradication of circulating polioviruses are overconfident at best. These results further support the Polio Eradication and Endgame Strategic Plan 2013–2018’s intention to withdraw all OPV vaccines by 2020 [2]. After withdrawal of OPV, worldwide reintroduction due to an outbreak should be carefully considered, as it appears a small amount of OPV may result in community transmission.

## Supplementary Data

Supplementary materials are available at *Clinical Infectious Diseases* online. Consisting of data provided by the authors to benefit the reader, the posted materials are not copyedited and are the sole responsibility of the authors, so questions or comments should be addressed to the corresponding author.

## Supplementary Material

Supplementary_Table_1

## Notes

***Acknowledgments***. The authors thank the families who participated in this study, the large field teams in Mexico that performed data and sample collection, as well as the broader team at Stanford University, including Christopher Anthony van Hoorebeke, Sean Leary, Aisha Talib, Jennifer Lee Kang, Rasika Behl, Meira Falkovitz-Halpern, ChunHong (Stacy) Huang, and Marvin Sommer.

***Financial support***. This work was supported by the Bill & Melinda Gates Foundation (grant number OPP1116910). C. I. J. was supported by the Medical Research Council Trials Methodology Hub (grant number MR/L004933/1- Q42).

***Supplement sponsorship.*** This article appears as part of the supplement “Polio endgame and beyond: Vaccine choices, transmission dynamics, and surveillance implications,” sponsored by The Bill & Melinda Gates Foundation.

***Potential conflicts of interest.*** C. I. J. received a grant from the Medical Research Council Trials Methodology Hub. All other authors report no potential conflicts. All authors have submitted the ICMJE Form for Disclosure of Potential Conflicts of Interest. Conflicts that the editors consider relevant to the content of the manuscript have been disclosed.
