# Supplementary material for: Spatial Analyses of Oral Polio Vaccine Transmission in an Community Vaccinated With Inactivated Polio Vaccine
Source: Clin Infect Dis. 2018 Oct 30;67(Suppl 1):S18–25. doi: 10.1093/cid/ciy622 (PMC6206123; doi:10.1093/cid/ciy622)
Supplement: Supplementary_Table_1 [file ciy622_suppl_supplementary_table_1.docx]

Supplementary Table 1: Spatial Characteristics comparing unvaccinated shedding with unvaccinated non-shedding individuals over time.

|  | Study Day |  |  |  |  |  |  |
| --- | --- | --- | --- | --- | --- | --- | --- |
|  | 1 | 4 | 7 | 10 | 14 | 21 | 28 |
| **70 % Coverage** |  |  |  |  |  |  |  |
| Vaccinated | 76 | 75 | 73 | 72 | 71 | 74 | 71 |
| Shedding | 25 (32·9%) | 50 (66·7%) | 46 (63·0%) | 34 (47·2%) | 17 (23·9%) | 12 (16·2%) | 7 (9·9%) |
| Unvaccinated | 133 | 128 | 127 | 126 | 124 | 121 | 124 |
| Shedding | 4 (3·0%) | 3 (2·3%) | 6 (4·7%) | 2 (1·6%) | 0 (0·0%) | 3 (2·5%) | 9 (7·3%) |
| Distance to nearest OPV Shedding Household | 69·6 (42·7, 112·0) | 64·7 (37·3, 82·4) | 58·4 (42·0, 85·0) | 68·7 (37·3, 103·2) | 133·6 (61·9, 217·1) | 132·3 (68·7, 176·1) | 128·5 (95·7, 292·8) |
| Number of OPV shedding within 100 Meters | 1 (0, 2) | 2 (1, 3) | 2 (1, 4) | 1 (0, 3) | 0 (0, 1·2) | 0 (0, 1) | 0 (0, 1) |
| Number of OPV shedding within 200 Meters | 4 (2, 5) | 7 (4, 9) | 7 (5, 10) | 6 (3, 8) | 2 (0, 4) | 2 (1, 3) | 1 (0, 2) |
| Number of OPV shedding within 500 Meters | 17 (13, 20) | 34 (29·5, 41) | 38 (28, 42) | 31 (21, 35) | 16 (13, 18) | 12 (7, 14) | 6 (4, 10) |
| Number of OPV shedding within 1000 Meters | 27 (26, 27) | 51 (50, 51) | 54 (53, 54) | 42 (41, 42) | 22 (22, 22) | 18 (16, 18) | 12 (12, 12) |
| **30 % coverage** |  |  |  |  |  |  |  |
| Vaccinated | 39 | 35 | 32 | 30 | 29 | 36 | 36 |
| Shedding | 13 (33·3%) | 19 (54·3%) | 21 (65·6%) | 14 (46·7%) | 8 (27·6%) | 11 (30·6%) | 10 (27·8%) |
| Unvaccinated | 342 | 306 | 299 | 291 | 306 | 316 | 307 |
| Shedding | 4 (1·2%) | 7 (2·3%) | 9 (3·0%) | 0 (0·0%) | 6 (2·0%) | 5 (1·6%) | 1 (0·3%) |
| Distance to nearest OPV Shedding Household | 107·9 (79·4, 180·5) | 97·7 (65·3, 160·7) | 88·2 (61·8, 162·9) | 118·3 (82·4, 235·5) | 157·3 (87·4, 257·4) | 160·7 (74·5, 262·3) | 136·6 (82·4, 203·3) |
| Number of OPV shedding within 100 Meters | 0 (0, 1) | 1 (0, 1) | 1 (0, 1) | 0 (0, 1) | 0 (0, 1) | 0 (0, 1) | 0 (0, 1) |
| Number of OPV shedding within 200 Meters | 2 (1, 3) | 3 (2, 4) | 3 (1, 5) | 2 (0, 3) | 1 (0, 2) | 2 (0, 3) | 1 (0, 3) |
| Number of OPV shedding within 500 Meters | 12 (8, 15) | 14 (9, 16) | 13 (8·5, 14) | 7 (6, 11) | 5 (4, 7) | 7 (4, 8) | 7 (4, 7) |
| Number of OPV shedding within 1000 Meters | 19 (19, 19) | 23 (23, 26) | 26 (23, 27) | 16 (14, 16) | 10 (9, 10) | 11 (11, 12) | 11 (11, 12) |
| **10 % coverage** |  |  |  |  |  |  |  |
| Vaccinated | 17 | 17 | 17 | 16 | 17 | 17 | 17 |
| Shedding | 6 (35·3%) | 10 (58·8%) | 9 (52·9%) | 10 (62·5%) | 4 (23·5%) | 4 (23·5%) | 2 (11·8%) |
| Unvaccinated | 560 | 546 | 556 | 555 | 558 | 560 | 550 |
| Shedding | 3 (0·5%) | 1 (0·2%) | 9 (1·6%) | 0 (0%) | 2 (0·4%) | 6 (1·1%) | 3 (0·5%) |
| Distance to nearest OPV Shedding Household | 259·9 (124·2, 441·2) | 134·7 (76, 194·3) | 138·8 (87·3, 197·6) | 134·7 (76·9, 194·3) | 238·9 (163·5, 363·9) | 241·3 (167·8, 363·9) | 395·6 (198·7, 732·2) |
| Number of OPV shedding within 100 Meters | 0 (0, 0) | 0 (0, 1) | 0 (0, 1) | 0 (0, 1) | 0 (0, 0) | 0 (0, 0) | 0 (0, 0) |
| Number of OPV shedding within 200 Meters | 0 (0, 1) | 1 (1, 2) | 1 (1, 2) | 1 (1, 2) | 0 (0, 1) | 0 (0, 1) | 0 (0, 1) |
| Number of OPV shedding within 500 Meters | 2 (1, 3) | 5 (3, 7) | 4 (2, 5·2) | 5 (3, 7) | 2 (1, 3) | 2 (1, 3) | 1 (0, 2) |
| Number of OPV shedding within 1000 Meters | 5 (5, 5) | 9 (8, 9) | 8 (7, 8) | 11 (10, 11) | 4 (3, 4) | 4 (3, 4) | 2 (2, 2) |
